# Retraction Note: *Vibrio parahaemolyticus* RhsP represents a widespread group of pro-effectors for type VI secretion systems

**DOI:** 10.1038/s41467-019-09094-0

**Published:** 2019-03-15

**Authors:** Nan Jiang, Le Tang, Ruiqiang Xie, Zhi Li, Brianne Burkinshaw, Xiaoye Liang, Dylan Sosa, L. Aravind, Tao Dong, Dapeng Zhang, Jun Zheng

**Affiliations:** 1 Faculty of Health Sciences, University of Macau, Macau SAR, China; 2 Department of Ecosystem and Public Health, University of Calgary, Calgary, AB T2N 4Z6 Canada; 3 Department of Biochemistry and Molecular Biology, University of Calgary, Calgary, AB T2N 4Z6 Canada; 4 Snyder Institute for Chronic Diseases, University of Calgary, Calgary, AB T2N 4Z6 Canada; 5 State Key Laboratory of Microbial Metabolism, Joint International Laboratory on Metabolic and Developmental Sciences, School of Life Sciences and Biotechnology, Shanghai Jiao Tong University, Shanghai, 200240 China; 6 Program of Bioinformatics and Computational Biology, College of Arts and Sciences, Saint Louis University, Saint Louis, MO 63103 USA; 7 National Center for Biotechnology Information, National Library of Medicine, National Institutes of Health, Bethesda, MD 20894 USA; 8 Department of Biology, College of Arts and Sciences, Saint Louis University, Saint Louis, MO 63103 USA

Retraction to: *Nature Communications;* 10.1038/s41467-018-06201-5; published online 25 Sep 2018

In this Article, we reported that, in *Vibrio parahaemolyticus*, secretion of protein RhsP by a Type VI secretion system requires removal of an N-terminal fragment by auto-proteolysis, followed by interaction with a PAAR protein.

However, it has come to our attention that parts of the experimental data reported in the paper were a result of image manipulation. Specifically, western blot images presented in Figs. 2e, 3a, 3b and 4c, and Supplementary Figs. 3a, 3b and 10, were subjected to manipulation including band duplication and rotation, cutting and pasting of bands into gels, and band elimination.

In light of these findings, we cannot be confident of the conclusions of the paper as it stands. We therefore wish to retract the paper. We deeply regret these circumstances and apologize to the scientific community. All authors agree with retraction of the Article.

